# High Levels of Within-Host Variations of Human Papillomavirus 16 *E1/E2* Genes in Invasive Cervical Cancer

**DOI:** 10.3389/fmicb.2020.596334

**Published:** 2020-11-24

**Authors:** Yusuke Hirose, Mayuko Yamaguchi-Naka, Mamiko Onuki, Yuri Tenjimbayashi, Nobutaka Tasaka, Toyomi Satoh, Kohsei Tanaka, Takashi Iwata, Akihiko Sekizawa, Koji Matsumoto, Iwao Kukimoto

**Affiliations:** ^1^Department of Obstetrics and Gynecology, Showa University School of Medicine, Tokyo, Japan; ^2^Department of Obstetrics and Gynecology, Faculty of Medicine, University of Tsukuba, Tsukuba, Japan; ^3^Pathogen Genomics Center, National Institute of Infectious Diseases, Tokyo, Japan; ^4^Department of Obstetrics and Gynecology, Keio University School of Medicine, Tokyo, Japan

**Keywords:** human papillomavirus, within-host variation, APOBEC, E1/E2, positive selection

## Abstract

Human papillomavirus type 16 (HPV16) is the most common HPV genotype found in invasive cervical cancer (ICC). Recent comprehensive genomics studies of HPV16 have revealed that a large number of minor nucleotide variations in the viral genome are present in each infected woman; however, it remains unclear whether such within-host variations of HPV16 are linked to cervical carcinogenesis. Here, by employing next-generation sequencing approaches, we explored the mutational profiles of the HPV16 genome within individual clinical specimens from ICC (*n* = 31) and normal cervix (*n* = 21) in greater detail. A total of 367 minor nucleotide variations (167 from ICC and 200 from the normal cervix) were detected throughout the viral genome in both groups, while nucleotide variations at high frequencies (>10% abundance in relative read counts in a single sample) were more prevalent in ICC (10 in ICC versus 1 in normal). Among the high-level variations found in ICC, six were located in the *E1/E2* genes, and all of them were non-synonymous substitutions (Q142K, M207I, and L262V for E1; D153Y, R302T, and T357A for E2). *In vitro* functional analyses of these E1/E2 variants revealed that E1/M207I, E2/D153Y, and E2/R302T had reduced abilities to support viral replication, and that E2/D153Y and E2/R302T failed to suppress the viral early promoter. These results imply that some within-host variations of *E1/E2* present at high levels in ICC may be positively selected for and contribute to cervical cancer development through dysfunction or de-stabilization of viral replication/transcription proteins.

## Introduction

Cervical cancer is the fourth most frequent cancer in women worldwide and is etiologically linked to persistent infections with high-risk genotypes of human papillomaviruses (HPVs) (zur Hausen, 2002). Of the approximately 15 high-risk HPVs, Human papillomavirus type 16 (HPV16) is most often detected in cervical cancer, indicating its strong potential for triggering cervical cancer development (Munoz et al., 2003). Incident infections with high-risk HPV in cervical mucosa clinically manifest as low-grade cervical intraepithelial lesions, which are generally eliminated within 1–2 years by the host immune response. However, in a subset of infected women, if the infection persists, the lesions can progress to a precancerous state, and eventually evolve into invasive cervical cancer (ICC) after more than a decade of viral persistence (Schiffman et al., 2007). Epidemiological evidence indicates that high-risk HPV infections can also be detected in healthy women with normal cervical cytology, reflecting a state of asymptomatic infection (de Sanjose et al., 2007).

Recent advances in next-generation sequencing technologies have facilitated a detailed understanding of viral genetic diversity within and between infected individuals (Cullen et al., 2015; de Oliveira et al., 2015; Mirabello et al., 2017; van der Weele et al., 2017; Dube Mandishora et al., 2018; Hirose et al., 2018; Lagstrom et al., 2019; Mariaggi et al., 2018). Although genomic sequences of DNA viruses are generally considered to be relatively stable compared to RNA viruses, HPV genomes within individual clinical specimens harbor a large number of minor genetic variants, so-called within-host genomic variability. In the HPV16 genome, these are mostly predominated by C-to-T or C-to-G substitutions, clearly implying the involvement of cellular APOBEC3 cytosine deaminases in their generation (Mirabello et al., 2017; Hirose et al., 2018). Such signature mutations are more frequently detected in normal or low-grade specimens of the cervix, suggesting that APOBEC3 mediates viral clearance by introducing deleterious mutations into the HPV16 genome (Hirose et al., 2018; Zhu et al., 2020). In contrast, in precancer/cancer specimens, some nucleotide variations in the HPV16 genome were found to be enriched to relatively high levels within a specimen (de Oliveira et al., 2015; Hirose et al., 2018; van der Weele et al., 2019). However, it is not clear whether such high-levels of variants are positively selected for, and whether they contribute to cervical cancer progression.

Here, by employing deep sequencing techniques, we focused our analysis on those within-host nucleotide variations in the HPV16 genome detected at high levels within individual specimens. Such high-level variations were more frequently found in ICC than in cytologically normal cervix. Of these, some non-synonymous variants in the *E1/E2* genes resulted in a decreased ability to support viral replication and transcription. Based on these results, we propose that selective pressure is being exerted on these *E1/E2* variants during cervical carcinogenesis.

## Materials and Methods

### Clinical Specimens

Our study subjects consisted of HPV16-positive women with normal cytology (NILM: negative for intraepithelial lesion or malignancy, *n* = 21) and ICC (*n* = 31), who visited the Keio University Hospital, Tsukuba University Hospital, or Showa University Hospital for cervical cancer screening or treatment of cervical diseases. Cervical smears were classified according to the Bethesda system. Histological diagnoses of ICC were made using hematoxylin and eosin sections of cervical biopsy specimens according to the World Health Organization classification. HPV16 positivity was determined by HPV genotyping as described previously (Azuma et al., 2014). The average age (±standard deviation) in each category was 37.1 (±11.4) for NILM and 41.6 (±12.9) for ICC (Supplementary Table). Using a cytobrush, exfoliated cervical cells were collected in ThinPrep (Hologic, Bedford, MA, United States) from the patients. Total cellular DNA was extracted from the cells on a MagNA Pure LC 2.0 (Roche Diagnostic, Indianapolis, IN, United States) using the MagNA Pure LC Total Nucleic Acid Isolation kit (Roche Diagnostic), and used for deep sequencing analyses of the HPV16 genome. The study protocol was approved by the ethics committees at each hospital and the National Institute of Infectious Diseases, and written informed consent for study participation was obtained from each patient.

### Detection of Nucleotide Variations in the HPV16 Genome

Overlapping PCR was performed with PrimeSTAR GXL DNA polymerase (Takara, Kusatsu, Japan) to cover the whole-genome sequence of HPV16. The sequences of PCR primers were as follows: HPV16-1744F (5′-TGT CTA AAC TAT TAT GTG TGT CTC CAA TG-3′) and HPV16-5692R (5′-GAT ACT GGG ACA GGA GGC AAG TAG ACA GT-3′); HPV16-5531F (5′-GGG TCT CCA CAA TAT ACA ATT ATT GCT G-3′) and HPV16-1980R (5′-TAT CGT CTA CTA TGT CAT TAT CGT AGG CCC-3′) (Hirose et al., 2018). The amplified DNA was subjected to agarose gel electrophoresis and purified using the Wizard gel purification kit (Promega, Madison, WI, United States). The purified DNA was converted to a DNA library using the Nextera XT DNA sample prep kit (Illumina, San Diego, CA, United States), followed by size selection with SPRIselect (Beckman Coulter, Brea, CA, United States). The multiplexed libraries were analyzed on a MiSeq (Illumina) with the MiSeq reagent kit v3 (150 cycle). Complete genomic sequences of HPV16 were assembled *de novo* from the total read sequences using the VirusTAP pipeline^1^ (Yamashita et al., 2016). The accuracy of the reconstructed whole-genome sequences was verified by read mapping with Burrows–Wheeler Aligner (BWA) v0.7.12 and subsequent visual inspection by Integrative Genomics Viewer (IGV) v2.3.90. Nucleotide mismatches compared to the assembled reference genome and positions of variations in each sample were identified using BWA and SAMtools v1.3.1 with in-house Perl scripts (available upon request). Based on a quality score confidence threshold of Phred quality score >30 (error probability <0.001) used to extract variation positions in the read sequences, we defined a position as heterogeneous if relative read abundance was >0.5% (Kukimoto et al., 2013). The presence of nucleotide substitutions was confirmed by manual inspection of mismatched read sequences using IGV.

### Transient Replication Assay

The HPV16 origin-containing plasmid and expression plasmids for N-terminal FLAG-tagged HPV16 E1 and E2 were described previously (Kukimoto et al., 2013). Expression plasmids for E1/E2 variants were constructed using the QuickChange Lightning Multi Site-Directed Mutagenesis Kit (Agilent Technologies, La Jolla, CA, United States). HPV-negative, cervical cancer C33A cells were plated 24 h before transfection in a 24-well plate at a density of 40,000 cells/well and transfected with 10 ng of the origin-containing plasmid for firefly luciferase expression and 10 ng of pGL4.75 (Promega) for *Renilla* luciferase expression together with 100 ng of the E1 expression plasmid and 50 ng of the E2 expression plasmid using the FuGENE HD reagent (Promega). The total quantity of transfected plasmid DNA was adjusted to 220 ng with the empty plasmid p3xFLAG-CMV10 (Sigma-Aldrich, St. Louis, MO, United States) as carrier DNA. At 72 h after transfection, firefly and *Renilla* luciferase activities were measured using the Dual-Glo Luciferase assay system (Promega) on an ARVO MX luminescence counter (PerkinElmer, Waltham, MA, United States), and the level of replication was quantified as the ratio of the two luciferase activities.

### Promoter Reporter Assay

The reporter plasmid containing the HPV16 early promoter, pGL3-P97, was described previously (Kukimoto et al., 2006). HPV18-positive, cervical cancer HeLa cells were plated 24 h before transfection in a 24-well plate at a density of 16,000 cells/well and transfected with 200 ng of pGL3-P97 or pGL3-Basic (Promega) and 5 ng of pGL4.75 with or without 40 ng of the E2 expression plasmid using the FuGENE6 reagent (Promega). The total quantity of transfected plasmid DNA was adjusted to 405 ng with p3xFLAG-CMV10. At 48 h after transfection, firefly and *Renilla* luciferase activities were measured as described above, and the level of transcription was quantified as the ratio of the two luciferase activities.

### Western Blot Analysis

C33A and HeLa cells were lysed on ice with RIPA buffer (20 mM Tris–HCl, pH8.0, 150 mM NaCl, 1 mM EDTA, 1 mM dithiothreitol, 1% Non idet P-40, and 5 mM MgCl_2_) supplemented with a protease inhibitor cocktail (Roche), followed by centrifugation at 21,000 × *g* at 4°C for 10 min. The cell lysates were separated by SDS-PAGE and transferred to PVDF membranes. The proteins were visualized by the ECL prime western blotting detection reagent (GE Healthcare, Chicago, IL, United States) with the following antibodies: anti-FLAG (M2; Sigma-Aldrich) and anti-α-tubulin (B-5-1-2; Sigma-Aldrich).

### Coimmunoprecipitation Assay

Human embryonic kidney 293 (HEK293) cells (2 × 10^6^ cells) were transfected with 5 μg of the E1 expression plasmids using FuGENE HD. At 48 h after transfection, total cell extracts were prepared in RIPA buffer as described above. N-terminal 6xHis-tagged HPV16 E2 (His-E2) was bacterially expressed and purified as previously described (Kusumoto-Matsuo et al., 2011). The cell extracts were incubated with 20 μg of His-E2 at 4°C for 2 h while mixing with anti-FLAG M2 magnetic beads (Sigma-Aldrich). The beads were washed three times with RIPA buffer, and the bound proteins were eluted by boiling the beads in SDS-PAGE sample buffer. The recovered proteins were analyzed by western blotting with anti-FLAG and anti-6xHis (HIS.H8; Abcam, Cambridge, United Kingdom) antibodies.

### DNA Pulldown Assay

Biotinylated DNA probes containing the HPV16 genomic region from 7,791 to 120 (236 base pairs in length) were prepared by PCR using the HPV16 whole-genome plasmid as a template with the following primers: HPV16-bio7791F (5′-biotin-TAC ATG AAC TGT GTA AAG GTT AGT CA-3′), and HPV16-120R (5′-TGT GGG TCC TGA AAC ATT GCA GTT CTC TTT-3′). The biotinylated DNA probes were coupled to Dynabeads/M-280 streptavidin (Dynal Biotech, Oslo, Norway) at room temperature for 20 min in coupling buffer (5 mM Tris–HCl, pH7.5, 0.5 mM EDTA, and 1 M NaCl). Total cell extracts were prepared from HEK293 cells that had been transfected with the E2 expression plasmids as described above, and incubated with the DNA-coupled or uncoupled magnetic beads at 4°C for 2 h. The beads were washed three times with RIPA buffer, and the bound proteins were eluted by boiling the beads in SDS-PAGE sample buffer and analyzed by western blotting with anti-FLAG antibody.

### Statistical Analysis

All statistical analyses were performed using R version 3.6.3.^2^ Mann–Whitney U test was used to evaluate the average variation number between the NILM and ICC samples. Welch’s *t*-test was used to evaluate a difference in variant frequency or reporter activity between different groups. A value of *p* < 0.05 was regarded as statistically significant.

### Data Availability

Short-read sequencing data are available from the DNA Data Bank of Japan, Sequence Read Archive, under accession number DRA009226.

## Results

### Within-Host Variations of HPV16 Genome Sequences in ICC and NILM

**FIGURE 1:**
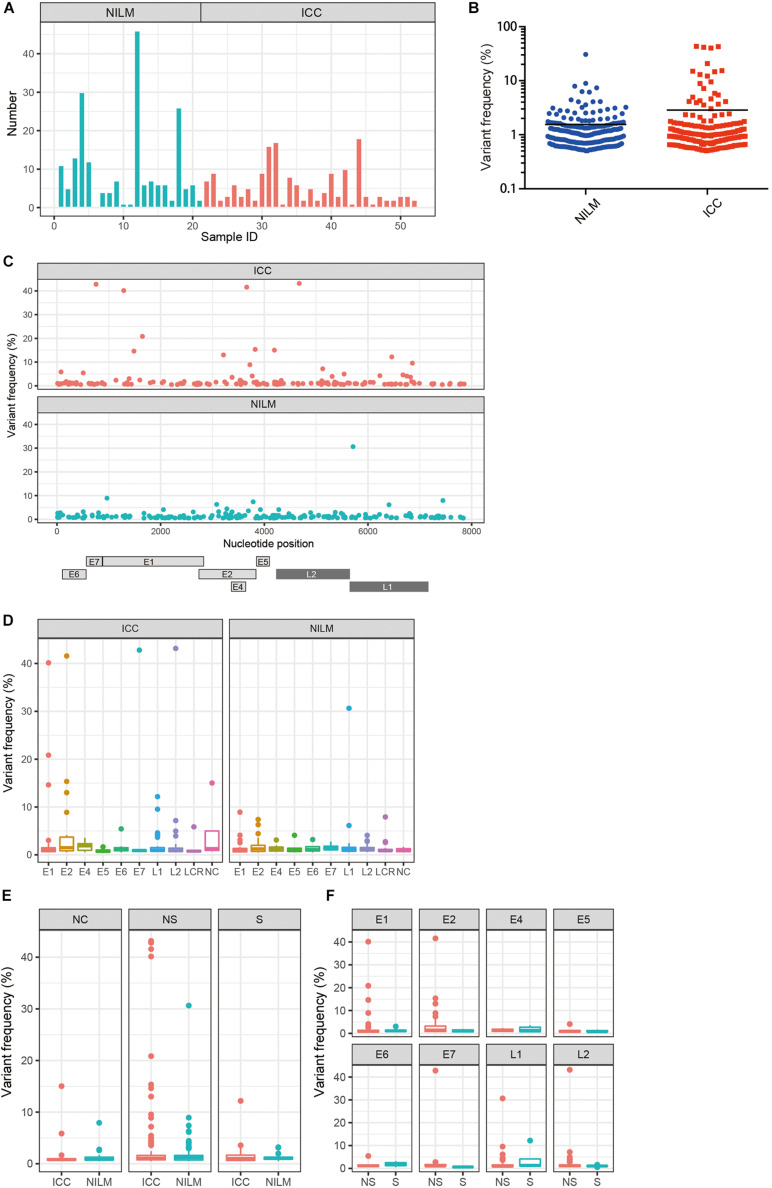
Within-host nucleotide variations across the HPV16 genome in negative for intraepithelial lesion or malignancy (NILM) and invasive cervical cancer (ICC). **(A)** Numbers of within-host nucleotide substitutions per sample (NILM, *n* = 21; ICC, *n* = 31). **(B)** Distribution of within-host frequencies of individual nucleotide substitutions in NILM and ICC samples. **(C)** Viral whole-genome landscape of within-host nucleotide substitutions present in NILM and ICC samples. Schematic representation of the HPV16 genome is shown below. **(D)** Boxplots of within-host frequencies of nucleotide substitutions in NILM and ICC according to the viral genomic regions. **(E)** Boxplots of within-host frequencies of nucleotide substitutions in NILM and ICC according to substitution types. NC, non-coding region between *E5* and *L2* and long control region; NS, non-synonymous substitution; S, synonymous substitution. **(F)** Boxplots of within-host frequencies of non-synonymous and synonymous substitutions according to the viral genomic regions.

Using our bioinformatics pipeline to detect minor nucleotide variations compared to a viral reference sequence dominantly present in each sample, we identified a total of 367 nucleotide substitutions in the HPV16 genome as within-host variations: 167 from ICC samples (*n* = 31) and 200 from NILM samples (*n* = 21) (Table 1). As we previously reported for another set of clinical samples (Hirose et al., 2018), non-synonymous substitutions far outnumber synonymous substitutions across all viral genes except *E4*. The number of variations per sample ranged from 1 to 18 in ICC (average, 5.4) and from 0 to 46 in NILM (average, 9.5) (Figure 1A). There was no significant difference in the average number of variations between the two groups (*p* = 0.20, Mann–Whitney U test). As shown in Figure 1B, the distribution pattern of within-host frequencies of individual variations was slightly different between ICC and NILM; nucleotide variations at relatively high frequencies were more apparent in ICC than in NILM. The average of variant frequencies was significantly different between the two groups (*p* = 0.02, Welch’s *t*-test). These nucleotide variations were almost completely evenly distributed throughout the viral genome in both NILM and ICC samples (Figure 1C), although a non-coding region between *E5* and *L2* (NC) showed the highest density of nucleotide variations (Table 1). When the variant positions were ranked according to viral genomic regions (*E1*, *E2*, *E4*, *E5*, *E6*, *E7*, *L1*, *L2*, LCR, and NC), it became apparent that the *E1*, *E2*, and *L2* regions contained more nucleotide variations at high frequencies in ICC than in NILM (Figure 1D). The *E7* and *L1* regions also harbored one exceptionally high-level variation in ICC and NILM, respectively.

**TABLE 1:** Numbers of nucleotide variations in the HPV16 genome.

Region	Length (bp)	Number	Density (/100 bp)	ICC	NILM	NS	S	p*
*E1*	1,950	76	3.9	35	41	63	13	0.60
*E2*	1,098	49	4.5	20	29	42	7	1
*E4*	288	17	5.9	6	11	10	7	0.01
*E5*	252	13	5.2	6	7	10	3	0.42
*E6*	456	28	6.1	14	14	26	2	0.40
*E7*	297	12	4.0	7	5	10	2	0.70
*L1*	1,596	58	3.6	29	29	54	4	0.14
*L2*	1,422	77	5.4	35	42	66	11	1
LCR	853	41	4.8	19	22	N/A	N/A	N/A
NC	134	16	11.9	4	12	N/A	N/A	N/A
Total	7,905	367	4.6	167	200	281	49	

NILM, negative for intraepithelial lesion or malignancy; ICC, invasive cervical cancer; NS, non-synonymous; S, synonymous; LCR, long control region; NC, non-coding region between E5 and L2; N/A, not applicable. *p-values for Fisher’s exact test comparing with the total distribution of NS and S.

We next compared the distribution of within-host frequencies of nucleotide variations between ICC and NILM based on three categories of nucleotide substitutions: non-synonymous, synonymous, and non-coding region substitutions. As shown in Figure 1E, high-level, non-synonymous substitutions were found more frequently in ICC than in NILM (*p* = 0.03, Welch’s *t*-test), whereas no such differential trend was apparent for synonymous and non-coding region substitutions (*p* = 0.56 for synonymous substitutions, and *p* = 0.43 for non-coding region substitutions, Welch’s *t*-test). The distributions of frequencies of non-synonymous versus synonymous substitutions were also examined according to individual gene regions. As shown in Figure 1F, the *E1*, *E2*, and *L2* regions were more enriched for non-synonymous substitutions at high frequencies (*p* = 0.16 for *E1*, *p* = 0.03 for *E2*, and *p* = 0.12 for *L2*, Welch’s *t*-test) compared to the other regions. The one *E7* and one *L1* variation detected at high levels (Figure 1D) were also non-synonymous substitutions.

### Patterns of Nucleotide Substitutions of Within-Host Variations

**FIGURE 2:**
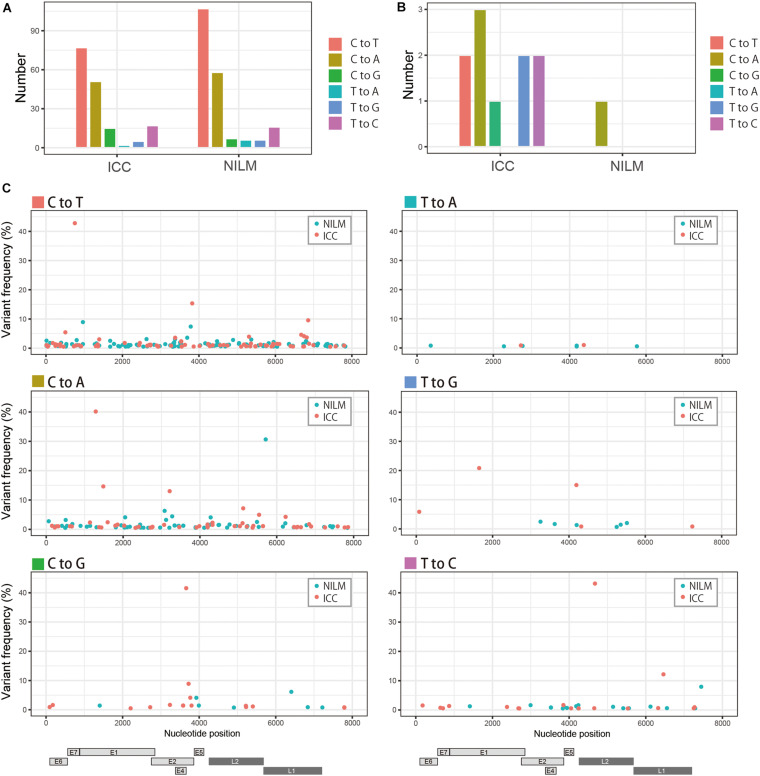
Patterns of within-host nucleotide substitutions in NILM and ICC. **(A)** Numbers of six types of nucleotide substitutions (C-to-T, C-to-A, C-to-G, T-to-A, T-to-G, and T-to-C) in NILM and ICC samples. **(B)** Numbers of six types of nucleotide substitutions (C-to-T, C-to-A, C-to-G, T-to-A, T-to-G, and T-to-C) at high frequencies (more than 10% abundance in relative read counts in a single sample) in NILM and ICC samples. **(C)** Viral whole-genome landscape of within-host nucleotide substitutions according to six types of substitutions (C-to-T, C-to-A, C-to-G, T-to-A, T-to-G, and T-to-C). Schematic representation of the HPV16 genome is shown below.

Recently, comprehensive genomics studies of HPV16 have documented that nucleotide variations in the HPV16 genome within individual clinical specimens were mostly C-to-T or G-to-A substitutions, which are believed to be mediated by cellular APOBEC3 cytosine deaminases as a host defense response to virus infection (Hirose et al., 2018; Zhu et al., 2020). We therefore examined the mutational spectrum in our clinical samples, based on six types of substitutions, i.e., C-to-A, C-to-G, C-to-T, T-to-A, T-to-C, and T-to-G (all substitutions are referred to by the pyrimidine of the mutated Watson–Crick base pair). As shown in Figure 2A, C-to-T substitutions were the most common in both ICC and NILM samples, followed by C-to-A substitutions. In contrast, the number of nucleotide variations at relatively high frequencies (>10%) exhibited a different pattern, and C-to-A substitutions were prevalent in ICC and NILM (Figure 2B). Overall, the C-to-T substitutions were evenly distributed across the HPV16 genome without any preference for specific viral regions regardless of sample histology (Figure 2C). No enrichment in particular regions was evident for the C-to-A substitutions as well as other minor types of substitutions.

### Within-Host Variations Present at High Levels

Highly abundant within-host variations, which we defined as nucleotide variants with >10% abundance in relative read counts in a single sample, were more frequently detected in ICC than in NILM: 10 variations in ICC and one variation in NILM (Table 2). Of these 11 high-level variations, 10 were located in coding regions (three in *E1*, three in *E2*, one in *E7*, two in *L1*, and one in *L2*), and only one in the non-coding region between *E5* and *L2*. Of the 10 coding-region variations, nine were non-synonymous or missense substitutions, generating variant forms of viral proteins: E1 Q142K, E1 M207I, E1 L262V, E2 D153Y, E2 R302T, E2 A357T, E7 L65F, L1 E52^∗^ (^∗^, nonsense mutation), and L2 N147D. Among these substitutions, E1 L262V, E2 D153Y, E2 R302T, and L1 E52^∗^ were not found by BLAST search, and thus were newly identified variations. Regarding the substitution pattern, as reflected in Figure 2B, C-to-A substitutions were dominant (4/11, 36%), followed by C-to-T (2/11, 18%), T-to-G (2/11, 18%), and T-to-C (2/11, 18%) substitutions.

**TABLE 2:** Highly abundant nucleotide variations in the HPV16 genome.

Sample ID	Position	Ref	Var	Abundance (%)	Region	Variant	Diagnosis
#22	4,674	A	G	43.2	*L2*	N147D^*a*^	ICC
#33	754	C	T	42.8	*E7*	L65F	ICC
#52	3,660	G	C	41.6	*E2*	R302T	ICC
#45	1,288	C	A	40.1	*E1*	Q142K	ICC
#15	5,712	G	T	30.6	*L1*	*52E^*a*^	NILM
#44	1,648	T	G	20.8	*E1*	L262V	ICC
#46	3,824	G	A	15.3	*E2*	A357T^*a*^	ICC
#45	4,195	T	G	15.0	NC	N/A	ICC
#30	1,485	G	T	14.6	*E1*	M207I	ICC
#35	3,212	G	T	13.0	*E2*	D153Y	ICC
#26	6,460	T	C	12.2	*L1*	N/A	ICC

NILM, negative for intraepithelial lesion or malignancy; ICC, invasive cervical cancer; N/A, not applicable. *Stop codon.^*a*^Variant corresponds to the prototype sequence.

### Biological Activities of E1/E2 Variants

The E1/E2 proteins play essential roles in viral replication; E2 recruits E1 to the viral origin DNA and E1 then unwinds the origin to initiate DNA replication. The enrichment of highly abundant, non-synonymous substitutions in the *E1/E2* genes prompted us to test whether the resulting E1/E2 variants had altered activities in supporting viral replication. To this end, the variant and prototype E1/E2 proteins were transiently expressed from expression plasmids in HPV-negative C33A cells, together with a viral origin-containing plasmid that included the firefly luciferase gene. Three days after transfection, replication levels of the origin plasmid were evaluated as a readout of elevated reporter activities. Although the transient replication assay does not reflect a replication mode of viral persistence, it has often been used to evaluate inherent activity of E1/E2 to induce viral replication. Two E1/E2 variants, E1 K483A (Nakahara et al., 2015) and E2 K111R (Thomas and Androphy, 2018), previously shown to be defective for viral replication, were included as negative controls. Because all E2 proteins so far reported have a threonine residue at position 357, we examined E2 T357A instead of A357T.

**FIGURE 3:**
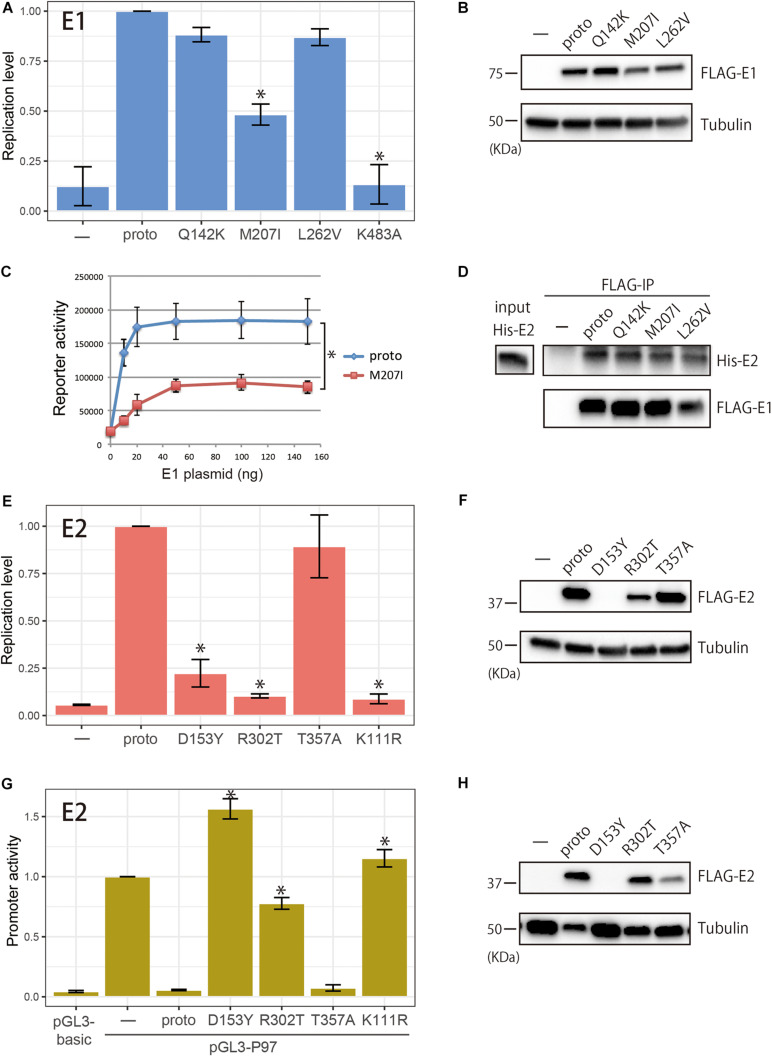
Biological activities of within-host variants of E1 and E2 proteins. **(A)** Replication activities of E1 variants. Expression plasmids (100 ng) for FLAG-tagged prototype or variant E1 proteins (Q142K, M207I, L262V, and K483A) were transfected into C33A cells together with the prototype E2 expression plasmid (50 ng), the HPV16 origin-containing firefly luciferase reporter plasmid (10 ng), and the *Renilla* luciferase plasmid (10 ng), and the levels of replication were measured 72 h after transfection. Relative replication levels compared to that of the prototype E1, which is set to 1.0, are shown. Error bars represent the standard deviation of three independent experiments. Statistically significant differences (Welch’s *t*-test, *p* < 0.05) are indicated with *. **(B)** Western blot analysis of E1 variants. FLAG-tagged prototype or variant E1 proteins expressed in C33A cells were detected with anti-FLAG antibody. Tubulin, loading control. **(C)** Titration of the E1 expression plasmids in the transient HPV16 replication assay. Increasing amounts of the E1 expression plasmid for the prototype E1 or M207 were transfected into C33A cells, and the levels of replication were measured 72 h after transfection. Relative light units of firefly luciferase activity divided by those of *Renilla* luciferase activity are shown. Error bars represent the standard error of two independent experiments. *Paired *t*-test (*p* < 0.05). **(D)** Coimmunoprecipitation of 6xHis-tagged E2 (His-E2) with the prototype and variant E1s. FLAG-tagged E1s were transiently expressed in HEK293 cells, and total cell extracts were prepared and mixed with recombinant His-E2, followed by immunoprecipitation with anti-FLAG magnetic beads. Immunoprecipitated proteins were analyzed by western blotting with anti-6xHis and anti-FLAG antibodies. Input, 10% of His-E2 used. **(E)** Replication activities of E2 variants. Expression plasmids (50 ng) for FLAG-tagged prototype or variant E2 proteins (D153Y, R302T, T357A, and K111R) were transfected into C33A cells together with the prototype E1 expression plasmid (100 ng), the HPV16 origin-containing firefly luciferase reporter plasmid (10 ng), and the *Renilla* luciferase plasmid (10 ng), and the levels of replication were measured 72 h after transfection. Relative replication levels compared to that of the prototype E2, which is set to 1.0, are shown. Error bars represent the standard deviation of three independent experiments. Statistically significant differences (Welch’s *t*-test, *p* < 0.05) are indicated with *. **(F)** Western blot analysis of E2 variants. FLAG-tagged prototype or variant E2 proteins expressed in C33A cells were detected with anti-FLAG antibody. Tubulin, loading control. **(G)** Transcription activities of E2 variants. Expression plasmids for FLAG-tagged prototype or variant E2 proteins (D153Y, R302T, T357A, and K111R) were transfected into HeLa cells together with the pGL3-P97 reporter plasmid, and transcription was measured 48 h after transfection. The promoter activity of pGL-P97 without E2 is set to 1.0. Error bars represent the standard deviation of three independent experiments. Statistically significant differences compared to the prototype E2 (Welch’s *t*-test, *p* < 0.05) are indicated with *. **(H)** FLAG-tagged prototype or variant E2 proteins expressed in HeLa cells were detected with anti-FLAG antibody. Tubulin, loading control.

Among the E1 variants tested, M207I had a significantly reduced ability to support replication of the origin plasmid relative to the prototype E1, whereas Q142K and L262V yielded similar levels of replication (Figure 3A). Western blot analysis revealed comparable levels of protein expression for the prototype and variant E1s (Figure 3B). In a titration experiment of the E1 expression plasmids, M207I showed a diminished ability to induce origin-dependent replication under the saturated amounts of the transfected plasmids when compared to the prototype (Figure 3C). Coimmunoprecipitation assay with FLAG-tagged E1s and 6xHis-tagged E2 demonstrated that all the E1 variants retained a capability to interact with E2 as the prototype E1 did (Figure 3D).

Regarding the E2 variants, D153Y and R302T exhibited a severely impaired ability to induce virus replication (Figure 3E). Transfection of increasing amounts of the E2 expression plasmids for D153Y and R302T also resulted in significantly reduced levels of replication compared to the prototype E2 (data not shown). Western blot analysis of the E2 variants showed considerable variability of protein levels (Figure 3F). In C33A cells, R302T was less efficiently expressed than the prototype E2, whereas the level of T357A was similar to that of the prototype. Although D153Y was almost undetectable on short exposure of the blot, longer exposure allowed the visualization of high molecular mass aggregates of this variant, which could not be resolved in the gel (data not shown), suggesting instability of D153Y in the cell. The defect of D153Y in viral replication was thus explained by a low expression level of this variant in C33A cells.

The E2 variants were further evaluated for their potential to regulate the viral early promoter in reporter assays, which was of interest because E2 is a known transcriptional repressor of the early promoter that drives *E6/E7* expression. HeLa cells were transfected with a reporter plasmid containing the HPV16 LCR upstream of the luciferase gene with or without the E2 expression plasmids, and 2 days after transfection, the reporter activity was measured. As shown in Figure 3G, while the prototype E2 completely suppressed the early promoter, D153Y and R302T failed to repress the viral promoter activity. In contrast, T357A suppressed the promoter activity as efficiently as the prototype E2 did. Western blot analysis showed comparable levels of protein expression for the prototype and R302T, and lower expression of T357A, whereas D153Y was almost undetectable as observed in C33A cells (Figure 3H).

### Spatial Location of D153 and R302 in the E2 Protein

**FIGURE 4:**
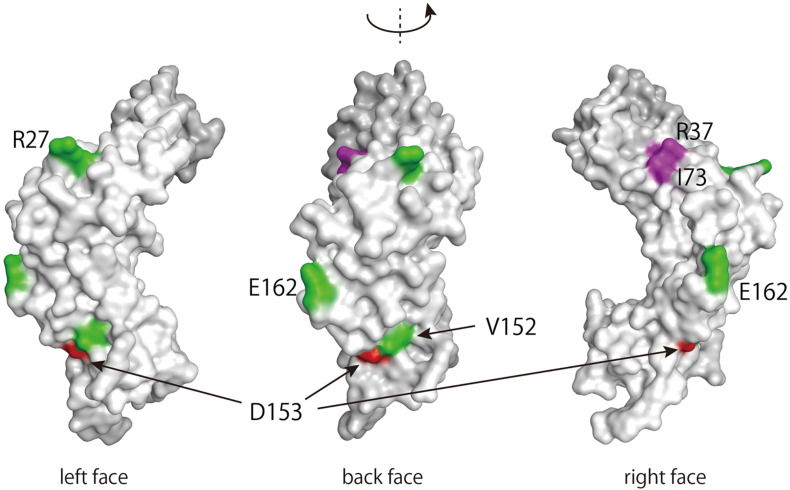
Three-dimensional structure of the transactivation domain of the HPV16 E2 protein. The transactivation domain structure of HPV16 E2 (PDB: 1DTO) was rendered in MOE (Molecular Operating Environment, Chemical Computing Group, Canada). Amino acid residues with within-host variations on the surface of the domain are colored red (D153) or green (R27, V152, and E162) (Mariaggi et al., 2018). Amino acid residues responsible for interacting with Brd4 (R37 and I73) are colored purple (McBride, 2013).

Finally, we determined the location of the variable amino acid residues of the E2 variants in the three-dimensional structure of the protein. The E2 protein is composed of three distinct domains: an N-terminal transactivation domain, a C-terminal DNA-binding domain, and a hinge-region connecting these two domains (McBride, 2013). Figure 4 shows a surface projection of the HPV16 E2 transactivation domain. Of its 201 amino acid residues, 176 are exposed at the surface, while 25 are buried inside the molecule. D153, which is included within this domain, is exposed at the molecular surface. Interestingly, V152 and E162, which were also shown to be mutated at high levels in a recent study (Mariaggi et al., 2018), are positioned spatially near D153.

**FIGURE 5:**
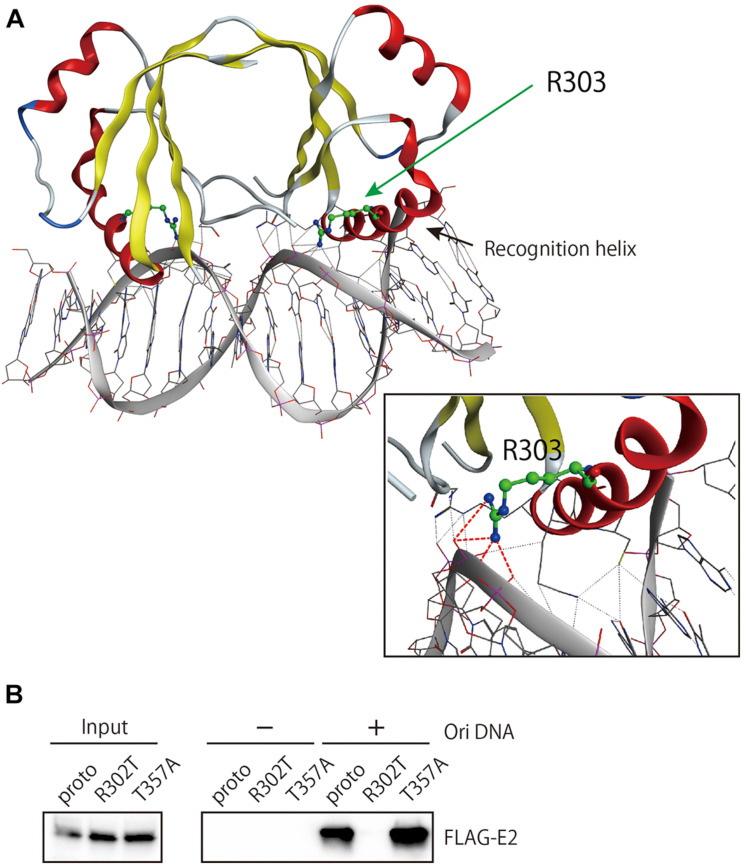
Origin DNA-binding activity of E2 variants. **(A)** The structure of the DNA-binding domain (DBD) of HPV18 E2 complexed with DNA (PDB: 1JJ4) was visualized in MOE. The spatial locations of R303, which is homologous to R302 in HPV16 E2, and the DNA recognition helix are shown. Hydrogen bonding between R303 and the DNA backbone is indicated as red dotted lines. **(B)** DNA pulldown assay for the prototype and variant E2 proteins. Total cell extracts prepared from HEK293 cells transfected with the E2 expression plasmids were mixed with magnetic beads coupled or uncoupled with the HPV16 origin DNA, followed by washing the beads. Bound proteins were analyzed by western blotting with anti-FLAG antibody. Input, 6% of cell extracts used.

The other two residues, R302 and T357, are included in the DNA-binding domain. Of note, R302 is highly conserved among E2 proteins from reported HPV genotypes and constitutes a key residue in the DNA recognition helix of E2 (McBride, 2013). In the DNA-binding domain of HPV18 E2 (Kim et al., 2000), the corresponding arginine residue makes direct contact with the phosphate backbone of DNA in the E2-binding sequence through hydrogen bonding (Figure 5A), indicating its direct role in interacting with DNA. Indeed, DNA pulldown assay using the viral origin DNA revealed that R302T completely lost binding activity to the origin, whereas T357A kept such activity as similarly as the prototype E2 did (Figure 5B).

## Discussion

Accumulating evidence indicates that HPV genomes often undergo mutagenesis in infected individuals, and APOBEC3 is a prime candidate for host proteins that generate such within-host viral genomic variability (Mirabello et al., 2017; Hirose et al., 2018; Mariaggi et al., 2018). APOBEC3 signature mutations in the HPV16 genome are more often detected in low-grade cervical lesions than in precancer/cancer samples, implying that APOBEC3 is involved in defending against HPV infections (Zhu et al., 2020). In the current study, we extended viral genomic analysis to asymptomatically HPV16-infected normal cervix and found that C-to-T substitutions, a typical pattern of APOBEC3 mutagenesis, were also dominantly detected in cytologically normal samples. This observation suggests that the host immune response mediated by APOBEC3 is operative even in asymptomatic infections and potentially contributes to viral clearance.

To further explore the biological significance of intra-host variability of the HPV16 genome, we focused on viral variations that were enriched at high levels within individual samples. Such high-level, single-nucleotide variations were more common among the ICC samples than the normal samples. This suggests that some selection process was responsible for their high level within a specimen because cervical cancer development requires a long period of HPV persistent infection. Consistent with such a selection scenario, the high-level nucleotide substitutions observed in the ICC samples were more enriched for non-synonymous substitutions, which strongly implies positive selection for particular intrahost variants of viral proteins.

Interestingly, the non-synonymous substitutions detected in ICC were more prominent in the *E1* and *E2* regions than in other regions of the viral genome. These two regions comprise relatively long open-reading frames (1,950 bp for *E1* and 1,098 bp for *E2*), but the length of a reading frame alone cannot explain the enrichment of *E1/E2* mutations because the late *L1* and *L2* regions of similar lengths (1,596 bp for *L1* and 1,422 bp for *L2*) harbored only one high-frequency non-synonymous substitution in *L1*. The enrichment of high-level, non-synonymous substitutions in *E1/E2* among the ICC samples prompted us to explore any functional changes in these gene products. Indeed, we found that of six E1/E2 variants tested, three (E1 M207I, E2 D153Y, and E2 R302T) had a reduced ability to regulate viral replication or transcription.

During cervical cancer progression, *E2* is often disrupted by integration into the host genome or transcriptionally silenced by epigenetic modifications (McBride and Warburton, 2017). Because the E2 protein negatively regulates the viral early promoter that drives *E6/E7* oncogenesis, functional loss of *E2* is thought to be strongly associated with the development of ICC via upregulation of *E6/E7*. Our finding that the two intra-host variants of E2 are defective for viral replication/transcription is consistent with the prevailing consensus on the role of E2 in HPV-induced carcinogenesis, implying the possibility that cells expressing D153Y and R302T had been positively selected for their contribution to cervical cancer development through enhanced expression of *E6/E7*.

A recent study including genomic analysis of HPV16 on serial cervical samples from precancer/cancer cases revealed that 56% of the women tested had an identical viral genomic sequence in two consecutive samples (the median time between sampling was 24 months) (Arroyo-Muhr et al., 2019). Moreover, the estimated substitution rate was almost zero substitutions/site/year, suggesting that HPV16 genomic sequences are extremely stable in most cases of persistent infections. However, another study with longitudinal sampling from primary and recurrent CIN2/3 lesions reported that of 14 paired samples, 10 had exactly the same sequences in consecutive samples, but 4 harbored relatively high-level nucleotide variations (5–50% abundance) in either the initial or follow-up samples (van der Weele et al., 2019). Interestingly, among six nucleotide positions detected as showing high-level variations in the CIN2/3 samples, four were located in the *E2* gene, and all of them were non-synonymous substitutions for the E2 protein (van der Weele et al., 2019). Enrichment of minor nucleotide variants in the *E2* gene was also demonstrated for HPV16-positive cervical specimens in another study (Mariaggi et al., 2018). These observations are consistent with our finding that the *E2* sequence is enriched in high-level, non-synonymous substitutions in cervical cancer samples and suggest that the enrichment of such *E2* variations is not a byproduct of cancer generation but precedes progression to ICC.

Among the E2 variants found in this study, D153Y seems to be less stably expressed in cells, which likely leads to the failure of this variant to regulate viral replication and transcription. Three-dimensional structural projection of the transactivation domain of E2 indicates that the D153 residue is exposed on the surface of the molecule, positioned spatially near residues that were previously reported to be subject to intrahost variation (Mariaggi et al., 2018). The cellular transcription factor Brd4 interacts with and stabilizes HPV16 E2 (Zheng et al., 2009), thereby supporting its transcriptional function. Although the Brd4-binding region includes R37 and I73 resides in the same transactivation domain of E2, it does not overlap at the surface with D153 (Figure 4). Because proteomics analysis of E2 revealed that a variety of cellular protein complexes interact with E2 (Jang et al., 2015), cellular proteins other than Brd4 might be involved in stabilizing E2 through interaction with the region around D153.

Consistent with the important role in DNA interaction suggested from structural inspection, R302T completely lost binding activity to the viral origin DNA containing three E2-recognition sites, and this defect nicely explains the inability of R302T to regulate viral origin-dependent replication and early promoter-driven transcription.

Regarding E1 variants, M207I showed a reduced ability to support viral origin-dependent replication, although it was expressed at a similar level to the prototype E1 and retained the ability to interact with E2. Such replication defect may be attributed to some change in DNA interaction during DNA unwinding by M207I because this residue is included in the DNA-binding domain of E1 (Auster and Joshua-Tor, 2004). Our previous study reported another within-host E1 variant, Q381E, which was present at a relatively high abundance (5.42%) in one ICC sample. This variant also exhibited a reduced ability to support HPV16 origin-dependent replication (Kukimoto et al., 2013). Expression of E1 induces a host DNA-damage response and causes growth arrest of the host cell (Fradet-Turcotte et al., 2011; Sakakibara et al., 2011). The E1 level is kept low through its proteasomal degradation induced by E1 itself to allow for virus persistence (Nakahara et al., 2015). Based on these observations, we hypothesize that functional attenuation of E1 may also favor the survival of infected cells and confer a selective growth advantage to those harboring such E1 variants.

Although our results suggest that the *E1/E2* genes are a hotspot for high-level within-host variations, it is also clear that not all the variations of E1/E2 cause functional changes or reduced expression. Because HPV-infected cervical lesions generally undergo two-way processes of progression and regression during a long period of persistent infection, it is very likely that a small viral population in the regression phases experiences a bottleneck for random selection of a minor variant genome (i.e., random genetic drift). Enrichment of variant viral genomes in cervical cancer specimens may reflect such neutral selection processes. In this regard, the presence of enriched nucleotide variations in the HPV genome is not a prerequisite but rather episodic for individual cases to eventually progress to ICC, as recently demonstrated for APOBEC3 mutagenesis in a range of human cancer cell lines (Petljak et al., 2019).

Nevertheless, highly abundant, non-synonymous variations in the *E1/E2* genes of cervical cancer specimens are reminiscent of the fact that most HPV-induced cancers present with viral integration into the host genome together with a breakpoint in *E1* or *E2* (McBride and Warburton, 2017). While our current study cannot distinguish between episomal and integrated forms of the viral genome, functional defects of E1/E2 caused by within-host variations may substitute for such integration events that are required for the development of ICC. Thus, consecutive monitoring of intra-patient *E1/E2* variations in precancerous lesions, such as CIN2/3, may contribute to clinical assessment of whether the lesions will progress if untreated. Further large-scale studies with longitudinal clinical samples will be needed to address this issue in more detail.

## Data Availability Statement

The datasets presented in this study can be found in online repositories. The names of the repository/repositories and accession number(s) can be found below: https://www.ddbj.nig.ac.jp/, DRA009226.

## Ethics Statement

The studies involving human participants were reviewed and approved by the Institutional Review Boards at Keio University Hospital, Tsukuba University Hospital, Showa University Hospital, and the National Institute of Infectious Diseases. The patients/participants provided their written informed consent to participate in this study.

## Author Contributions

IK conceived and designed the study, and wrote and revised the manuscript. YH and YT obtained the sequencing data. MY-N, KT, and IK performed *in vitro* experiments. YH, YT, MY-N, and IK analyzed and interpreted the data. MO, NT, TS, TI, AS, and KM collected the clinical specimens. All authors read and approved the final manuscript.

## Conflict of Interest

The authors declare that the research was conducted in the absence of any commercial or financial relationships that could be construed as a potential conflict of interest.

## Footnotes

1 https://gph.niid.go.jp/cgi-bin/virustap/index.cgi

2 https://cran.r-project.org/
