# Supplementary material for: High Levels of Within-Host Variations of Human Papillomavirus 16 E1/E2 Genes in Invasive Cervical Cancer
Source: Front Microbiol. 2020 Nov 24;11:596334. doi: 10.3389/fmicb.2020.596334 (PMC7721666; doi:10.3389/fmicb.2020.596334)
Supplement: Supplementary file 1 [file Table_1.DOCX]

**Supplementary Table. HPV16-positive clinical samples analyzed in this study**

| ID | Diagnosis | Age (year) | Variation (*n*) | Other HPV |
| --- | --- | --- | --- | --- |
| 1 | NILM | 27 | 11 | HPV51/52 |
| 2 | NILM | 38 | 5 | HPV52/58 |
| 3 | NILM | 32 | 13 | - |
| 4 | NILM | 34 | 30 | HPV51 |
| 5 | NILM | 32 | 12 | HPV31/54/68 |
| 6 | NILM | 49 | 0 | - |
| 7 | NILM | 44 | 4 | - |
| 8 | NILM | 27 | 4 | - |
| 9 | NILM | 69 | 7 | HPV44 |
| 10 | NILM | 35 | 1 | - |
| 11 | NILM | 38 | 1 | - |
| 12 | NILM | 24 | 46 | HPV18/59 |
| 13 | NILM | 62 | 6 | - |
| 14 | NILM | 38 | 7 | HPV31 |
| 15 | NILM | 37 | 6 | - |
| 16 | NILM | 33 | 6 | - |
| 17 | NILM | 43 | 2 | HPV66 |
| 18 | NILM | 30 | 26 | HPV58 |
| 19 | NILM | 32 | 5 | - |
| 20 | NILM | 31 | 6 | - |
| 21 | NILM | 25 | 2 | - |
| 22 | SCC | 35 | 7 | - |
| 23 | SCC | 33 | 9 | - |
| 24 | SCC | 36 | 2 | - |
| 25 | SCC | 46 | 3 | - |
| 26 | SCC | 36 | 6 | - |
| 27 | SCC | 29 | 3 | - |
| 28 | SCC | 35 | 5 | - |
| 29 | SCC | 36 | 2 | - |
| 30 | SCC | 47 | 9 | - |
| 31 | SCC | 35 | 16 | - |
| 32 | SCC | 37 | 17 | - |
| 33 | SCC | 34 | 1 | - |
| 34 | SCC | 29 | 8 | - |
| 35 | SCC | 64 | 6 | - |
| 36 | SCC | 29 | 2 | - |
| 37 | SCC | 39 | 5 | - |
| 38 | SCC | 37 | 2 | - |
| 39 | SCC | 80 | 4 | - |
| 40 | SCC | 41 | 9 | - |
| 41 | SCC | 44 | 3 | - |
| 42 | SCC | 41 | 10 | - |
| 43 | SCC | 33 | 1 | - |
| 44 | SCC | 33 | 18 | - |
| 45 | SCC | 73 | 3 | - |
| 46 | SCC | 60 | 1 | HPV56 |
| 47 | SCC | 38 | 3 | - |
| 48 | SCC | 32 | 2 | - |
| 49 | AC | 38 | 2 | - |
| 50 | AC | 29 | 3 | - |
| 51 | PC | 57 | 3 | - |
| 52 | NC | 53 | 2 | - |

NILM, negative for intraepithelial lesion or malignancy; SCC, squamous cell carcinoma; AC, adenocarcinoma; PC, poorly differentiated carcinoma; NC, neuroendocrine carcinoma.
